# The Diseases of Infancy and Childhood

**Published:** 1902-11

**Authors:** 


THE DISEASES OF INFANCY AND CHILDHOOD. For the Use of Students and Practitioners in Medicine. By L. EMMETT HOLT, M. D., LL. D., Professor of Diseases of Children in the College of Physicians and Surgeons (Columbia University), New York; attending Physician to the Babies' and Foundling Hospitals. Octavo, pp. 1178. Illustrated. Second edition, revised and enlarged. New York: D. Appleton & Co. 1902. [Price, cloth, $6.00; half-leather, $6.50.]

When Holt's work first appeared five years ago it at once took rank as one of the leading American textbooks on diseases of children. The second edition has been revised and many of its chapters in part rewritten, much new matter, both in the way of text and illustration being added. In the main the revision has been carefully done. It is regretable, however, that the chapter on infant feeding continues in part an error of the first edition. A colored plate facing p. 163 aims to show in a graphic way the composition of various proprietary foods as compared with woman's milk, the dry foods for purposes of analysis being each mixed with a certain definite proportion of water. It is manifestly unfair to analyse in this way such foods as Mellin's and Imperial granum which are intended to be used with milk and do not, as do the others, contain milk in the dry state. A much more satisfactory analysis of proprietary foods is given in Louis Fischer's recent work on Infant Feeding. M. J. F.

